# Supplementary figures and images for: Disc-shaped fossils resembling porpitids or eldonids from the early Cambrian (Series 2: Stage 4) of western USA
Source: PeerJ. 2017 Jun 6;5:e3312. doi: 10.7717/peerj.3312 (PMC5463991; doi:10.7717/peerj.3312)

Electron Image 5

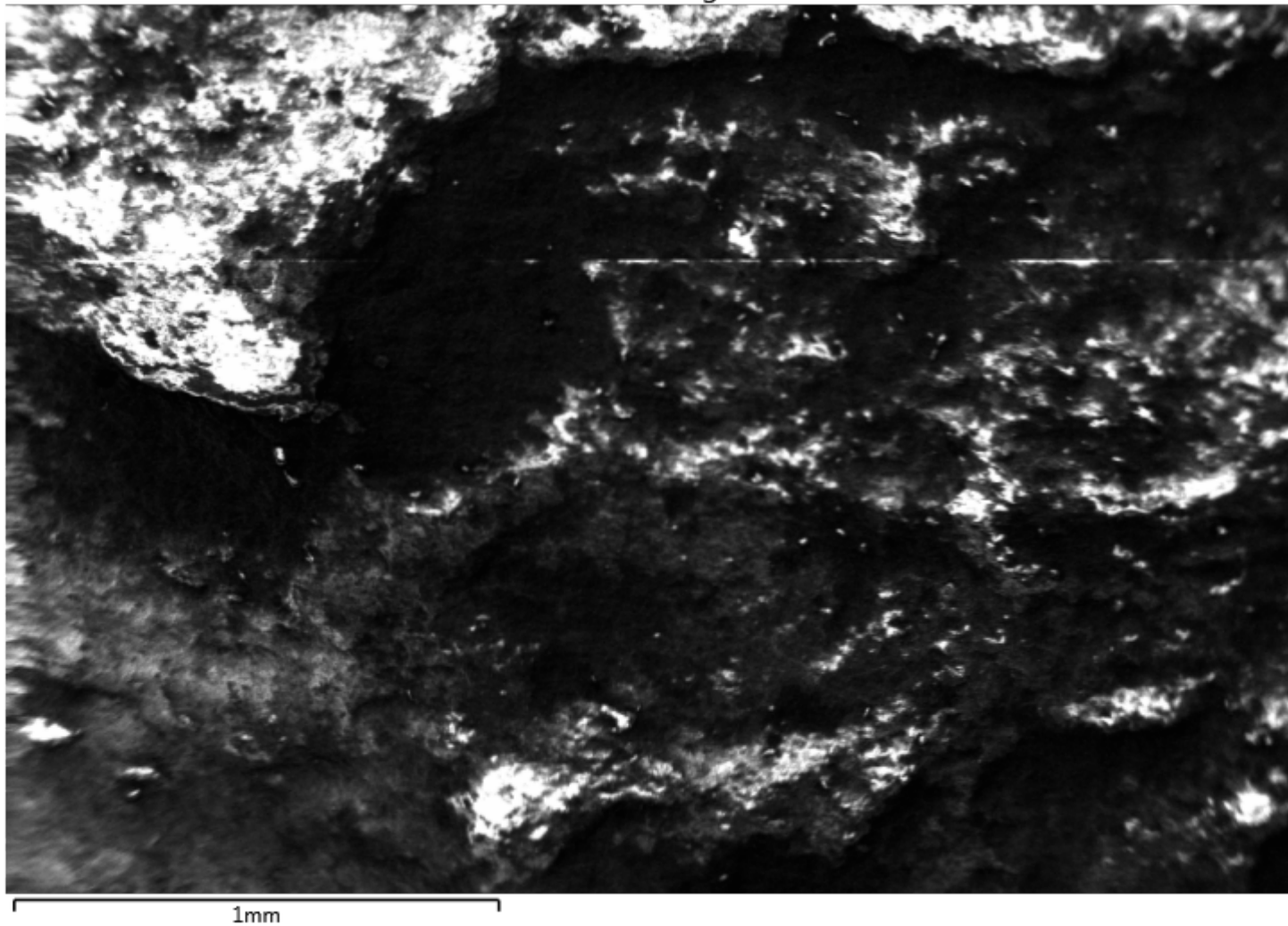

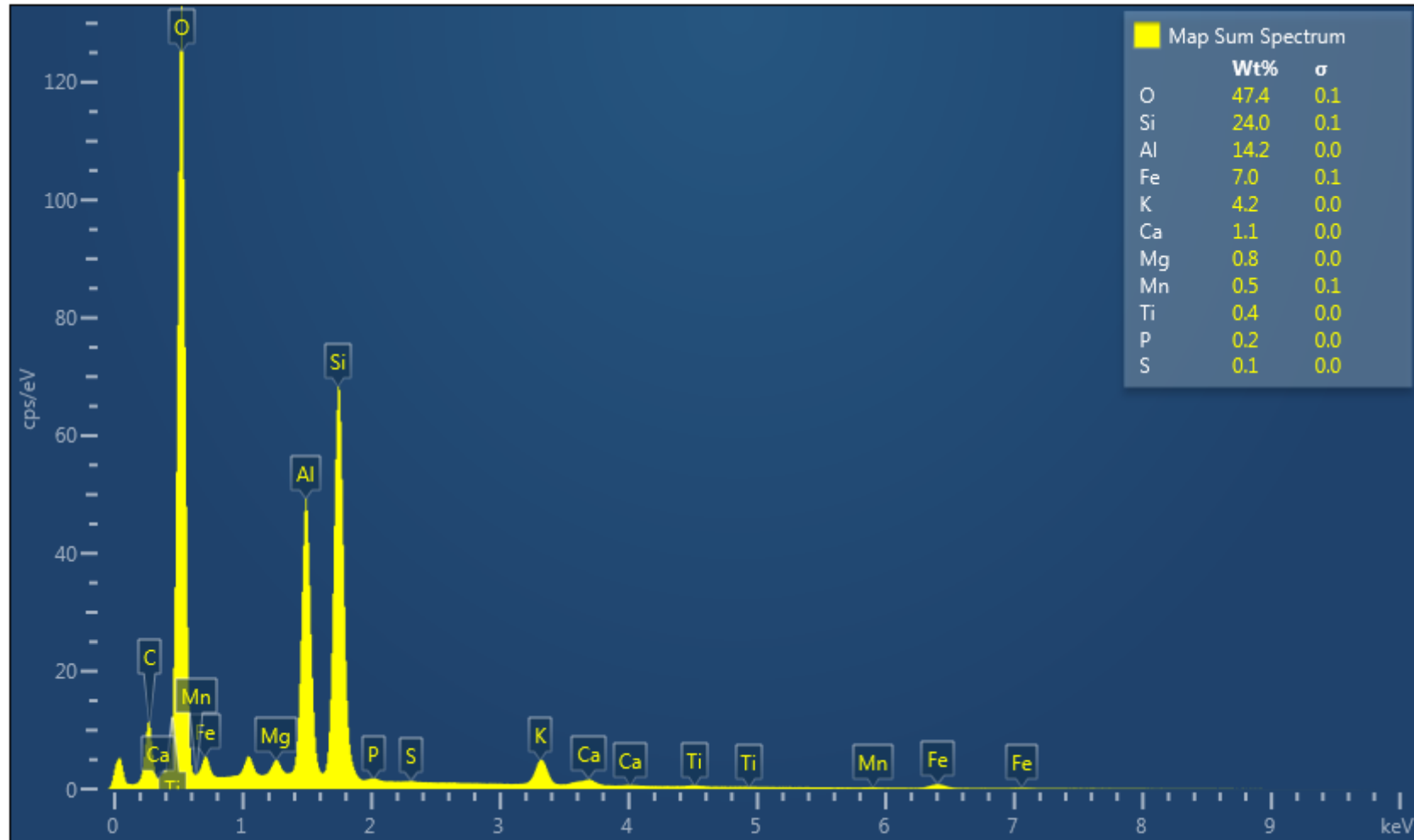

Supplement: Supplemental Information 1 — An SEM image and the spectra and weight percentages of elements for the portion of the Discophyllum fossil, KUMIP specimen 389538, in the region demarcated by the blue box labeled “Fig. 4” in Fig. 3A; maps shown in Fig. 4. [file peerj-05-3312-s001.pdf]

Electron Image 8

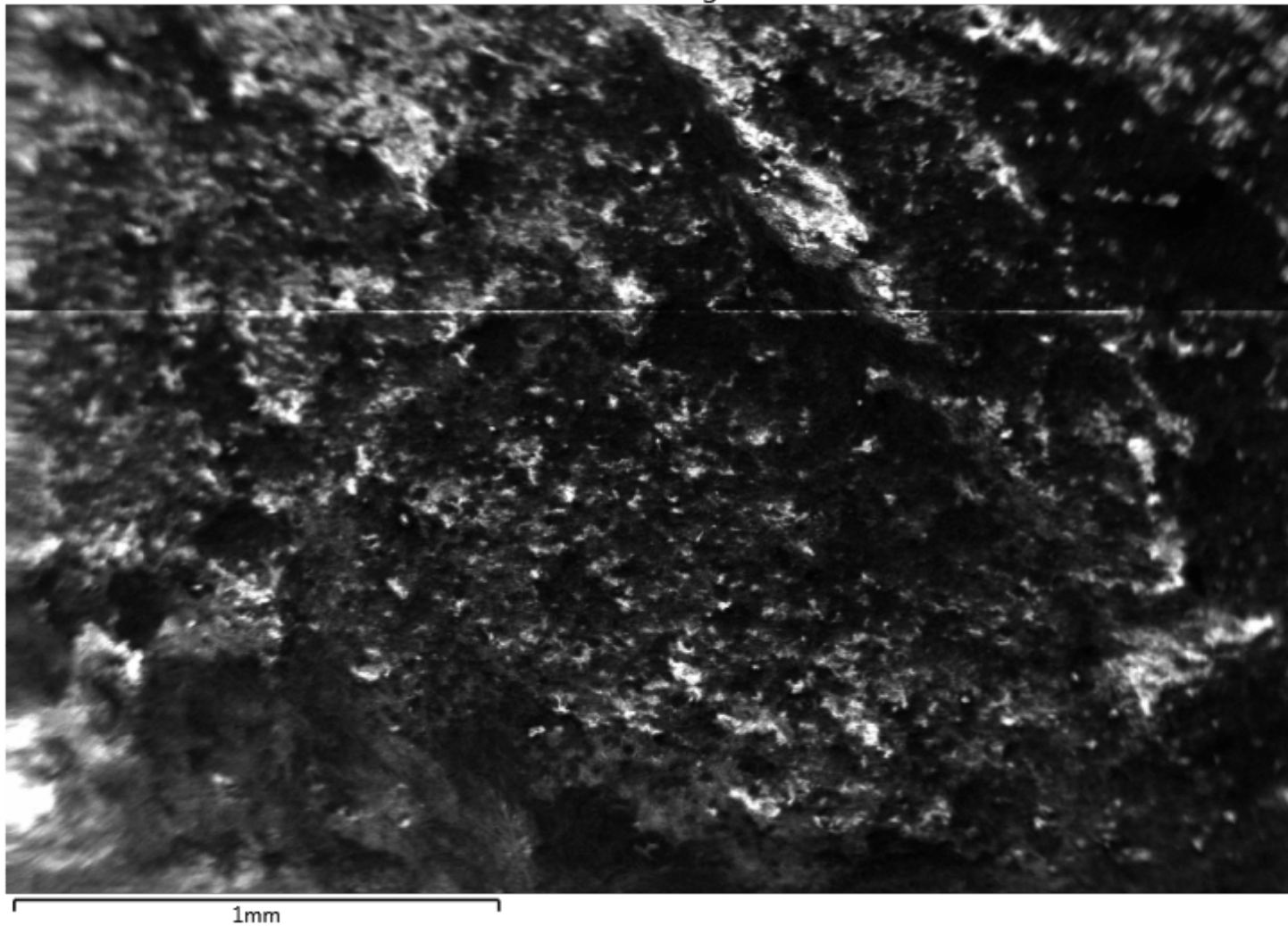

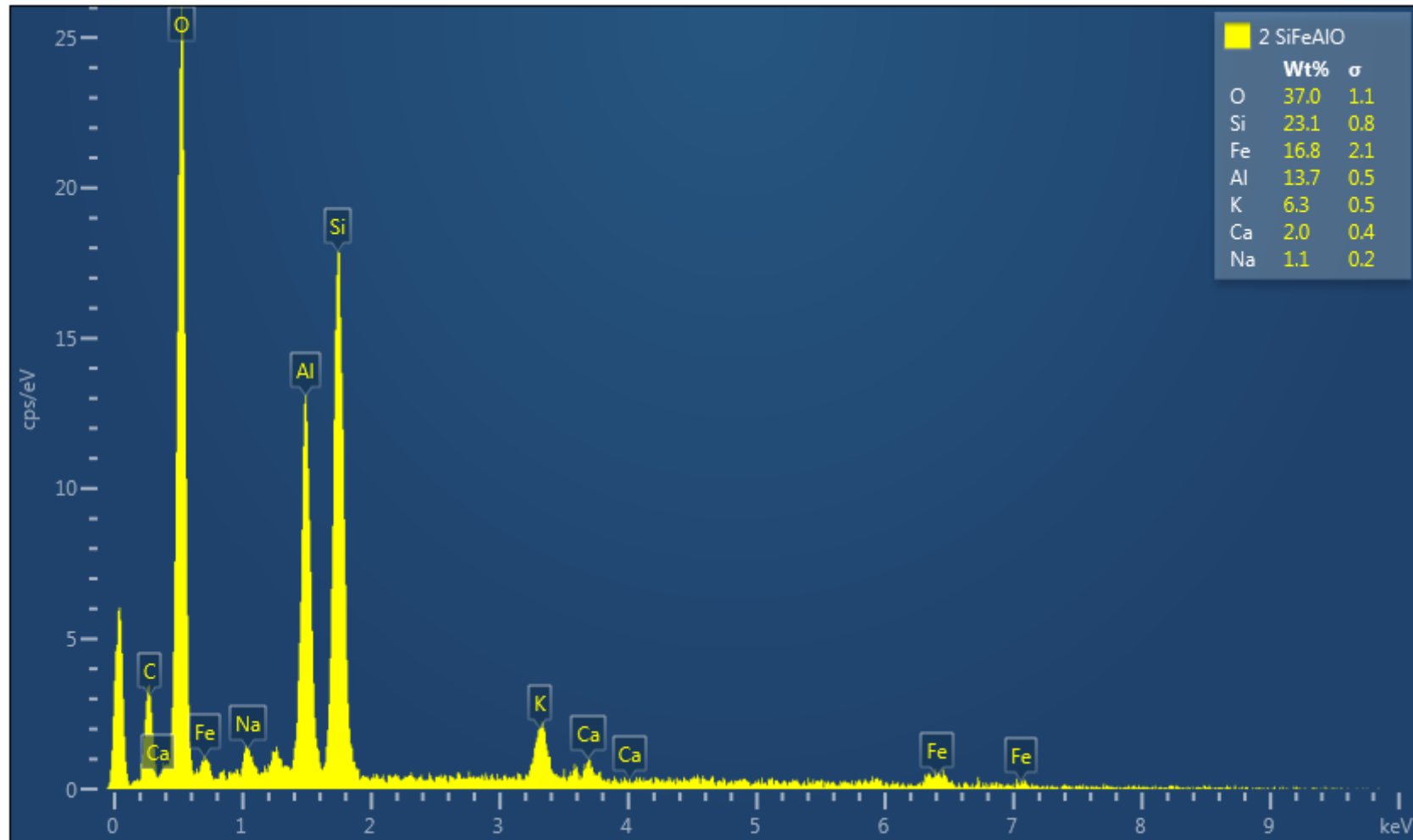

Supplement: Supplemental Information 2 — An SEM image and the spectra and weight percentages of elements for the portion of the Discophyllum fossil, KUMIP specimen 389538, in the region demarcated by the blue box labeled “Fig. 5” in Fig. 3A; maps shown in Fig. 5. [file peerj-05-3312-s002.pdf]
